# High Prevalence of Hypertension in Ethiopian and Non-Ethiopian HIV-Infected Adults

**DOI:** 10.1155/2018/8637101

**Published:** 2018-01-29

**Authors:** Maya Korem, Tali Wallach, Michael Bursztyn, Shlomo Maayan, Karen Olshtain-Pops

**Affiliations:** ^1^AIDS Unit, Department of Clinical Microbiology and Infectious Diseases, Hadassah-Hebrew University Medical Center, Jerusalem, Israel; ^2^Department of Pediatrics, Kaplan Medical Center, Rehovot, Israel; ^3^Hypertension Unit, Department of Medicine, Hadassah-Hebrew University Medical Center, Mount Scopus, Jerusalem, Israel; ^4^Infectious Diseases and Infection Control Unit, Barzilai Medical Center, Ashkelon, Israel

## Abstract

**Objectives:**

Prevalence of hypertension has not been studied in the Ethiopian HIV-infected population, which represents 60% of the patients in our AIDS unit. Our aim was to identify risk factors and characterize the prevalence of hypertension in the population monitored at our unit.

**Methods:**

A retrospective chart review categorized subjects according to their blood pressure levels. Hypertension prevalence was determined and stratified according to variables perceived to contribute to elevated blood pressure.

**Results:**

The prevalence of hypertension in our study population was significantly higher compared to the general population (53% versus 20%, *P* < 0.0001) and was associated with known risk factors and not with patients' viral load and CD4 levels. Ethiopian HIV-infected adults had a prominently higher rate of blood pressure rise over time as compared to non-Ethiopians (*P* = 0.016).

**Conclusions:**

The high prevalence of hypertension in this cohort and the rapid increase in blood pressure in Ethiopians are alarming. We could not attribute high prevalence to HIV-related factors and we presume it is part of the metabolic syndrome. The lifelong cardiovascular risk associated with HIV infection mandates hypertension screening and close monitoring in this population.

## 1. Introduction

The increased use of highly active antiretroviral therapy (HAART) in recent decades has led to a reduction in morbidity and mortality among people living with Human Immunodeficiency Virus (HIV) and has modified the course of HIV to a chronic infection with long-term complications, one of which is cardiovascular disease (CVD) [1, 2]. Among HIV-infected individuals, CVD is a main cause of morbidity and mortality, comprising 10% of non-AIDS-related mortalities [3].

The increased risk of CVD is attributed to a combination of factors. Prolonged viremia is associated with elevated systemic inflammatory markers [4–6], hypercoagulation, damage to the endothelium [7], and premature atherosclerosis [8]. Prolonged HAART administration is associated with a linear increase in cardiovascular-related mortality for up to 5 years of HAART exposure [9, 10]. This increase is related to the occurrence of lipodystrophy and metabolic derangements such as elevated cholesterol and triglyceride levels [11], insulin resistance, and impaired glucose tolerance [9, 10, 12].

Fifty percent of coronary heart disease in developed countries is attributed to hypertension (HTN) [13]. HTN is increased in subjects with diabetes and dyslipidemia, which are more prevalent among HIV-infected individuals than in the general population [3, 14, 15], and has become a potentially significant cause of morbidity in this population [16]. HTN in HIV-infected adults is associated with a higher frequency of persistent proteinuria, coronary heart disease, and myocardial infarction compared to nonhypertensive HIV-infected subjects [17].

The reported prevalence of HTN in HIV-infected adults shows great variance. It has been described as ranging from 8% to 34% [18, 19] and was not found to be consistently higher than the levels found in matched HIV-negative adults [20]. An increased incidence of HTN was observed in HIV-infected adults treated with HAART for 48 weeks, as compared with HIV-infected HAART-naïve adults (26% versus 7%, resp.) [19]. In a recent meta-analysis of seven hypertension studies, the prevalence of abnormal diurnal blood pressure (BP) pattern that may contribute to the high CVD risk ranged from 29 to 82% among HIV-infected individuals, compared to 15–53% in their HIV-negative counterparts [21].

Ethnic origin is a known factor affecting BP. Numerous studies conducted in the US found increased incidence and severity of HTN in African Americans compared with Caucasians [22, 23]. The Ethiopian population is Semitic in origin and not frequently studied in HTN trials. A large meta-analysis of HTN in Equatorial African countries, which included studies from 1998 to 2008, estimated the incidence of HTN in Ethiopia as 10.6% [24]. In a study conducted in young Ethiopian immigrants in Israel, BP was significantly higher among young male Ethiopian immigrants compared with a group of Israeli students, despite a dramatically lower body mass index (BMI). In addition, the prevalence of HTN was higher in Ethiopian immigrants after 2 years of residence in Israel, compared to <3 months' residency in the same boarding schools [25].

The HIV unit at the Hadassah Medical Center is unique in that Ethiopian immigrants constitute 60% of the patients monitored in the clinic. This high rate allows for a HTN evaluation study in this population.

The goal of this retrospective study was to estimate the prevalence of elevated blood pressure (BP) among our cohort of HIV-infected adults and to compare its prevalence in Ethiopians and non-Ethiopians. In addition, we aimed to identify risk factors for the occurrence of elevated BP. To our knowledge, no previous studies have yet focused on BP in Ethiopian HIV-infected adults.

## 2. Methods

### 2.1. Study Population

The observational analytical cross-sectional study was based on data retrieved from medical records of HIV-infected adults aged ≥18 years, who had been followed up at the AIDS unit in the outpatient clinic of the Hadassah (Ein Karem) Medical Center during the years 2000–2012. This adult HIV center, the only one in Jerusalem, monitors approximately 400 HIV-infected adults every 3 months. Each visit includes BP measurement, routine blood tests (including viral load and CD4 levels), an interview, and a physical examination.

As a control to our study population, we used BP data from a survey conducted by the Ministry of Health and the Central Bureau of Statistics in Israel during 2003-2004, which included approximately 10,000 participants (Supplement 1).

The research protocol for this study was approved by the Research Ethics Committee of the Hadassah-Hebrew University Medical Center.

### 2.2. Blood Pressure Measurement, Definition, and Collection

Blood pressure (BP) was measured at each visit in the clinic using a digital blood pressure machine (General Electric, DINAMAP ProCare 120). Measurements were taken by the staff nurse, after at least 5 minutes of rest, on the right upper arm, in the sitting position. BP levels were categorized into four levels, based on the seventh report of the Joint National Committee (JNC7) [26]: normal (systolic BP < 120 mm Hg, diastolic < 80 mm Hg); prehypertension (systolic BP 120–139 mm Hg, diastolic BP 80–89 mm Hg); stage 1 HTN (systolic BP 140–159 mm Hg, diastolic BP 90–99 mm Hg), and stage 2 HTN (systolic BP ≥ 160 mm Hg, diastolic BP ≥ 100 mm Hg). The term HTN was generally used in this work for patients classified as HTN stage 1 or 2 as measured during two separate visits in the clinic over the follow-up period, or when antihypertensive medication was used for treating hypertension regardless of BP level in the clinic.

BP data from four time points during follow-up were collected from patient files: close to HIV diagnosis, at the time of the lowest recorded CD4 count, at the time the highest BP was recorded, and at the last visit.

### 2.3. Clinical Data Collection and Risk Factor Definitions

Clinical and laboratory data were obtained from the medical records. Basic clinical data included age, gender, ethnic origin, height, antiretroviral treatment, duration of HIV infection from the date of diagnosis, CVD risk factors, and family history of CVD. Laboratory data included CD4 cell counts, categorized as <200, 200–349, 350–500, and >500 cells/*μ*l, and HIV-1 RNA or viral load, categorized as undetectable, ≤1000, >1000–10,000, >10,000–100,000, ≥100,000 copies/ml, HDL cholesterol, triglycerides, and fasting plasma glucose. Weight and all laboratory data were recorded at the same 4 time points as BP data. BMI ≥ 25 kg/m^2^ was defined as overweight. Metabolic syndrome was defined according to the criteria of the National Cholesterol Education Program (NCEP) Adult Treatment Panel III (ATP III) 2005 [27]. In cases where diabetes mellitus had not been diagnosed, we used ADA criteria for the diagnosis [28]. Tobacco use was defined as any current or previous use. Family history of CVD was defined as development of atherosclerotic CVD in a first-degree relative younger than 55 (males) or 65 (females).

### 2.4. Statistical Methods

All statistical analyses were conducted using SPSS software version 21.0. Statistical significance was assessed at the 0.05 level, and 2-sided *P* values are reported.

One-sample chi-square test was used to compare the prevalence of hypertension in the study group and in the general population. The *t*-test was used to compare quantitative variables between two independent groups. The association between two categorical variables was tested by a chi-square test and Fisher's exact test as appropriate.

The correlation between two quantitative variables was estimated by calculating the Pearson correlation coefficient.

Variables found to have a statistically significant association with the dependent variable (HTN) in a univariate analysis were examined in a multivariate model of logistic regression. To test which factors had impact on blood pressure over a period of time, a variability analysis model with repeated measurements was used.

## 3. Results

The study cohort consisted of 297 patients living with HIV, monitored at the Hadassah AIDS Center between January 2000 and December 2012. Within this group, 257 (86%) patients were on HAART according to accepted guidelines [29].

### 3.1. Study Population Characteristics

Two hundred and seventy-eight subjects were included in the final analysis, representing 94% of the patients followed up at the clinic during the study period. The remaining 19 patients did not have sufficient follow-up information in their files. Mean age of study participants was 42 (range: 22–80 years). Most subjects were of Ethiopian origin (172/278, 61.8%). Overall, 157 (56%) were men, but the proportion of men was significantly lower in Ethiopian (73/172, 42%) than in non-Ethiopian (84/106, 79%) patients (*P* < 0.001). Mean CD4 count at baseline for all participants was 350 ± 255 cells/ml; 30% had a CD4 count below 200 cells/ml. Mean follow-up time was 7 years.

### 3.2. Prevalence of HTN

Fifty-three percent (147/278) of the subjects were categorized as having HTN according to the highest recorded BP, which is significantly higher than the 20% prevalence in the general Israeli population, according to the national health survey conducted by the Central Bureau of Statistics (CBS) in 2003-2004 (*P* < 0.0001). This discrepancy remained significant when gender and age (21–44 and ≥45 years) were stratified. BP data from the last visit in the clinic showed a lower prevalence of HTN, 63/278 (22.6%), which is similar to the prevalence in the general population.

HTN rate was similar in Ethiopian and non-Ethiopian HIV-infected subjects (93/172, 54% versus 54/106, 51%, resp.).

### 3.3. Prevalence of HTN by Stratified Characteristics and Risk Factors

Table 1 describes the stratification of participants' characteristics according to three levels of blood pressure (normal, pre-HTN, and HTN) that were measured at two follow-up time points: (1) when the highest BP was measured and (2) at the last visit in the clinic.

The prevalence of HTN was significantly higher in males than in females. Subjects with metabolic syndrome or diabetes showed a greater propensity for HTN (94.5% and 87.5%, resp.; *P* < 0.001), as did obese (68.8%, *P* < 0.01) or older (73.6%, *P* < 0.001) participants.

However, when multivariate analysis was used, the only factor that associated significantly with HTN was the presence of metabolic syndrome (*P* < 0.001).

### 3.4. Prevalence of HTN by Disease Parameters

No significant association was found between viral load or CD4 levels measured at nadir and HTN (*P* > 0.05) (Table 2).

The duration of HIV before HTN diagnosis was estimated using 2 parameters: (1) the duration of time measured between HIV diagnosis and maximal BP measurement and (2) the CD4 level at nadir, which is considered an indirect measure of disease duration. Both parameters had no significant effect on the prevalence of HTN.

### 3.5. Impact of Ethnic Origin and the Time Elapsed from HIV Diagnosis to the Highest Recorded BP on the Prevalence of HTN

The highest BP was recorded on average 7 years (range: 1–20 years) after HIV diagnosis. The mean maximal BP was 141/83, significantly higher than that measured at baseline (122/77, *P* < 0.001).

No significant correlation was found between duration of the period since HIV diagnosis to the highest recorded BP, or to HTN diagnosis, and there was no significant difference in HTN prevalence between Ethiopian and non-Ethiopian HIV-infected subjects between these two time points. However, we found a significant interaction between time and ethnic origin: the rate of diastolic and systolic BP rise over time was significantly higher in Ethiopian than in non-Ethiopian HIV-infected subjects (*P* = 0.016).

## 4. Discussion

This study describes the prevalence of HTN in HIV-infected adults in an urban setting in Israel, with a focus on patients of Ethiopian origin, a population that has not been thoroughly studied.

Prevalence of HTN in all study subjects was significantly higher among HIV-infected adults (53%) compared to the general population (20%, *P* < 0.0001). Male gender and age were significantly associated with HTN, as reported for non-HIV populations [30]. This high prevalence was found at the point of the highest recorded BP, as well as at CD4 nadir. This finding is in concordance with results from a study conducted in Italy [31], which showed that the incidence of HTN is significantly higher among HIV-infected adults, compared to a control cohort of healthy blood donors (34.2% and 11.2%, resp.). The authors suggest that the higher resistance to insulin found among HIV-infected adults as compared with the controls (HOMA index 1.2 ± 3.3 versus 0.9 ± 2, resp.; *P* < 0.001) could explain this finding. In addition, higher HOMA indices were found among HIV-infected adults with HTN as opposed to those without HTN (3.7 ± 1 versus 1.2 ± 3.1, *P* < 0.001).

Other studies have also reported high rates of insulin resistance among HIV-infected adults treated with HAART [32, 33]. Hyperinsulinemia contributes to HTN by several mechanisms, including increased effect of angiotensin II on aldosterone and vascular contraction [34]. It is also linked to the metabolic syndrome, a strong independent cardiovascular risk factor [35] with increased prevalence among HIV-infected adults [36]. Insulin resistance was also predictive of the development of hypertension in lean, young Ethiopian immigrants in Israel [37].

A correlation between HAART and increased prevalence of HTN in HIV-infected adults has been reported [31, 34]. In a study conducted in Spain [19], which included patients who began HAART, a statistically significant rise in systolic and diastolic BP was found after 48 weeks of treatment. However, we did not find a significant association between HAART and the presence of HTN. The lack of agreement among the studies may be related to the inclusion of HIV-infected adults with initially lower CD4 values in other studies [19]. Commencing HAART in these subjects may have led to weight gain and consequently to HTN. Another explanation is the low rate of HIV-infected adults that were not treated with HAART in our study.

We found obesity, family history, advanced age, diabetes, and metabolic syndrome to be associated with HTN. These are the known risk factors for HTN, closely related to insulin resistance in the general population [38], and therefore support the reliability of our findings. Yet, we could not point to a clear reason for the higher prevalence of HTN in this population. Possibly, the HIV associated chronic inflammatory process is responsible for an increased prevalence of the metabolic syndrome in HIV patients and for the higher prevalence of HTN found in this cohort, which we assume is part of the metabolic syndrome.

A recent study [39] found nadir CD4 values below 50 cells/*μ*l to be an independent risk factor for the development of HTN in HIV-infected adults. Possible explanations for these results are inflammation of the small intestine found at low CD4 levels (HIV enteropathy) with activation of the immune system and release of inflammatory mediators, contributing to elevation of angiotensin II and salt accumulation [40, 41]. We did not find nadir CD4 values to be associated with a high prevalence of HTN, possibly because we categorized CD4 counts to higher values, which were chosen according to the guidelines for starting HAART therapy [29].

In our cohort, no association was found between viral load or CD4 levels at nadir and the presence of HTN. Both parameters are indirect measures of disease duration and do not accurately reflect the duration of viremia.

Unlike results from other studies [22, 23], no significant correlation was found between ethnic origin and the presence of HTN. However, the rate of BP increase over time was significantly higher in Ethiopian than in non-Ethiopian HIV-infected subjects. This finding may be related to lifestyle and dietary modifications as a result of immigration and is supported by a research conducted in Israel among Ethiopian adolescents, which pointed to the impact of an adopted new lifestyle on BP [25]. It is important to note that in this study the proportion of male patients of Ethiopian origin was significantly lower than in the non-Ethiopian group (42% versus 79%, *P* < 0.001). Since male patients had a significantly higher prevalence of HTN, this may have resulted in an underestimation of HTN prevalence among patients of Ethiopian origin.

A limitation of this study resides in its retrospective nature, which prevents determining the cause of HTN. Current guidelines define HTN as the average of two or more properly measured readings at each of two or more office visits after an initial screen [26]. With multiple measurements, high BP tends to decrease during subsequent visits [26, 42]. However, elevated BP may be predictive of future BP elevations [43], and the high prevalence of HTN was recorded at two different time points under identical conditions during the whole follow-up period. This somewhat reduces the probability of inconsistency between measurements. Although HTN data obtained at the last visit was similar to the values in the general population (63/278, 22.6%), this may have been the result of antihypertensive medication provided to patients with high BP during follow-up.

## 5. Conclusions

HIV-infected adults have an increased risk of cardiovascular morbidity and related risk factors, such as the metabolic syndrome.

Our study reports increased prevalence of HTN in this population in comparison with the general population and points to an increased rate of BP rise in Ethiopian HIV-infected adults. Despite the lack of a clear explanation for this finding, we postulate that prolonged replication of the virus and the resulting inflammatory process may lead to metabolic syndrome and related hypertension. Raising awareness of the treating physicians to the significance of this risk factor will allow better monitoring and earlier treatment, reducing the impact of this risk factor on cardiovascular mortality.

## Figures and Tables

**Table 1:** Participants' characteristics by three levels of BP at two time points of follow-up.

Number of patients (% of total cohort)						
Characteristics	Highest BP			Last visit BP		
	Normal	Pre-HTN	HTN	Normal	Pre-HTN	HTN
Ethnicity						
Ethiopian	12 (7)	67 (39)	93 (54)	59 (34.3)	73 (42.4)	40 (23.2)
Non-Ethiopian	2 (1.9)	50 (47.2)	54 (51)	33 (31.1)	50 (47.2)	23 (21.7)
			*P* = 0.2			*P* = 0.87

Gender						
Female	9 (7.4)	61 (50.4)	51 (42.2)	51 (42.1)	50 (41.3)	20 (16.5)
Male	5 (3.2)	56 (35.7)	96 (61.2)	41 (26.1)	73 (46.4)	43 (27.3)
			*P* < 0.01			*P* < 0.05

Age (years)						
21–44	10 (5.4)	94 (51)	80 (44.1)	74 (39.5)	77 (41.1)	36 (19.2)
≥45	4 (4.2)	23 (24.4)	67 (71.2)	18 (19.7)	46 (50.5)	27 (29.6)
			*P* < 0.001			*P* < 0.01

Overweight						
No	5 (7.1)	33 (47.1)	32 (45.7)	29 (42)	26 (37.7)	14 (20.2)
Yes	0 (0)	15 (31.3)	63 (68.8)	8 (16.7)	24 (50)	16 (33.3)
			*P*^1^ = 0.01			*P*^1^ = 0.01

Metabolic syndrome						
No	5 (5.4)	46 (50)	41 (44.5)	33 (36.3)	44 (48.4)	14 (15.4)
Yes	0 (0)	1 (5.6)	17 (94.5)	0 (0)	6 (33.3)	12 (66.7)
Diabetes mellitus	0 (0)	1 (12.5)	7 (87.5)	2 (25)	2 (25)	4 (50)
			*P* < 0.001			*P*^1^ < 0.001

Family history of CVD						
No	5 (4.9)	45 (43.7)	53 (51.4)	34 (33.3)	46 (45.1)	22 (21.5)
Yes	0 (0)	4 (23.5)	13 (76.5)	2 (11.8)	6 (35.3)	9 (53)
			*P*^1^ = 0.26			*P*^1^ = 0.02

Tobacco use						
No	1 (3.1)	16 (50)	15 (46.8)	14 (43.8)	13 (40.6)	5 (15.6)
Yes	5 (5.6)	35 (38.9)	50 (55.5)	25 (28.1)	39 (43.8)	25 (28.1)
			*P*^1^ = 0.3			*P*^1^ = 0.41

CD4 count (cells/*µ*l)						
≤200 (*n* = 131)	8 (6.1)	51 (38.9)	72 (54.9)	46 (34.3)	56 (42.7)	30 (23)
>200 (*n* = 147)	6 (4)	66 (44.8)	75 (51)	46 (29.9)	67 (47.6)	33 (22.4)
			*P* = 0.64			*P* = 0.64

Use of HAART						
No (*n* = 38)	4 (10.8)	16 (43.2)	17 (45.9)	15 (33.3)	22 (48.8)	8 (17.7)
Yes (*n* = 240)	10 (4.1)	101 (41.9)	130 (53.9)	77 (33)	101 (43.3)	55 (23.6)
			*P* = 0.38			*P* = 0.69

^1^Fisher's exact test was performed for low prevalence; BP: blood pressure; HTN: hypertension; CVD: cardiovascular disease; HAART: highly active antiretroviral treatment.

**Table 2:** Prevalence of HTN measured at the time of the lowest recorded CD4 count according to categorized CD4 and viral load levels (*P* > 0.05).

	Number of patients (% of total cohort)		Total
	BP status		
	No HTN	HTN	
CD4 count (cells/*µ*l)			
<200	70 (47.9)	76 (52)	146 (100)
200–349	37 (43.5)	48 (56.5)	85 (100)
350–499	15 (50)	15 (50)	30 (100)
≥500	9 (53)	8 (47)	17 (100)
Total	131 (47.1)	147 (52.8)	278 (100)

VL^1^ (copies/ml)			
<50	23 (40.4)	34 (59.6)	57
50–1000	9 (39.1)	14 (60.9)	23
>1000–10,000	16 (42.1)	22 (57.9)	38
>10,000–100,000	34 (54.8)	28 (45.2)	62
>100,000	31 (48.4)	33 (51.6)	64
Total	113	131	244 (100)

^1^Data was missing for *n* = 34; BP: blood pressure; HTN: hypertension; VL: viral load.

## Supplementary Materials

Supplementary Materials Supplement 1: blood pressure data from a survey conducted by the Ministry of Health and the Central Bureau of Statistics in Israel during 2003-2004.
