# Supplementary material for: High Prevalence of Hypertension in Ethiopian and Non-Ethiopian HIV-Infected Adults
Source: Int J Hypertens. 2018 Jan 29;2018:8637101. doi: 10.1155/2018/8637101 (PMC5830020; doi:10.1155/2018/8637101)
Supplement: Supplementary Materials — Supplement 1: blood pressure data from a survey conducted by the Ministry of Health and the Central Bureau of Statistics in Israel during 2003-2004. [file 8637101.f1.pdf]

Supplement 1. Blood pressure data from a survey conducted by the Ministry of Health and the Central Bureau of Statistics in Israel during 2003-2004**.**

Morbidity

Chronically ill
By illness, age, gender
And population group
Rate per 100 persons

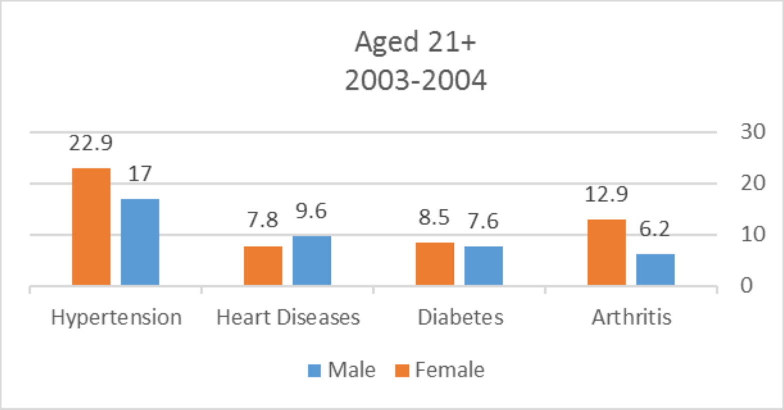

	Hypertension	Heart Diseases	Diabetes	Arthritis
**Total**	**20**	**8.7**	**8.1**	**9.7**
21-44	6.8	1.8	2.4	4.1
45-64	27.4	10.7	11.2	12.3
65+	51.1	28.2	21.7	23.7
**Gender**				
**Males**	**17**	**9.6**	**7.6**	**6.2**
21-44	5.6	2	1.7	3.3
45-64	26.1	13.4	11.8	8
65+	42.5	31.6	22.1	13.7
**Females**	**22.9**	**7.8**	**8.5**	**12.9**
21-44	8.1	1.6	3.1	4.9
45-64	28.6	8.3	10.6	16.2
65+	57.7	25.6	21.4	31.4
**Population group**				
**Jews and others**	**20.7**	**9**	**8.1**	**9.2**
21-44	6.8	1.9	2.5	3.6
45-64	26.6	10	10.3	10.9
65+	52	28.6	21.4	22.9
**Arabs**	**15.2**	**6.3**	**7.7**	**12.9**
21-44	7	(1.3)	(2)	6.4
45-64	34.8	17.8	19.6	25.5
65+	34.9	(20.2)	27.6	39

Source: Health Survey 2003-2004, Ministry of Health and CBS
Based on reported ever having been diagnosed with the chronic illness.
( ) Estimate for which the relative sampling error is 25%-40%, or estimates based on 11-20 respondents.
